# Public Health Impacts of Ambient Particulate Matter Pollution in Libya from 1990 to 2019: An Analysis of the 2019 Global Burden of Disease (GBD) Study

**DOI:** 10.3390/ijerph21060667

**Published:** 2024-05-23

**Authors:** David Rojas-Rueda, Sandhya Lamsal, Mohini Kak, Sameh El-Saharty, Christopher H. Herbst

**Affiliations:** 1 Department of Environmental and Radiological Health Sciences, Colorado State University, Environmental Health Building, 1601 Campus Delivery, Fort Collins, CO 80523, USA; sandhya.lamsal@colostate.edu; 2 Colorado School of Public Health, Colorado State University, Environmental Health Building, 1601 Campus Delivery, Fort Collins, CO 80523, USA; 3 Health, Nutrition and Population Global Practice, The World Bank, Riyadh 11432, Saudi Arabia

**Keywords:** ambient air pollution, PM_2.5_, burden of disease, Libya, DALY

## Abstract

Air pollution is recognized as a critical global health risk, yet there has been no comprehensive assessment of its impact on public health in Libya until now. This study evaluates the burden of disease associated with ambient particulate matter (PM_2.5_) in Libya, drawing on data from the Global Burden of Disease Study 2019. By integrating satellite-based estimates, chemical transport models, and ground-level measurements, PM_2.5_ exposure and its effects on mortality and disability-adjusted life years (DALYs) across the different sexes and all age groups from 1990 to 2019 are estimated. Our findings reveal that the annual population-weighted mean PM_2.5_ concentration in Libya was 38.6 μg/m^3^ in 2019, marking a 3% increase since 1990. In the same year, PM_2.5_ was responsible for approximately 3368 deaths, accounting for 11% of all annual deaths in the country. Moreover, a total of 107,207 DALYs were attributable to PM_2.5_, with ischemic heart disease being the leading cause, representing 46% of these DALYs. The analysis also highlights a significant burden of years of life lost (YLLs) at 89,113 and years lived with disability (YLDs) at 18,094, due to PM_2.5_. Given the substantial health risks associated with air pollution, particularly from ambient particulate matter, Libyan authorities must implement effective policies aimed at reducing air pollution to enhance healthcare outcomes and preventive services.

## 1. Introduction

Air pollution is a complex mixture of liquid, gases, and particulate matter (PM). Black carbon, organic aerosol, sulfate (SO_4_^2−^) nitrate (NO_3_^−^), ammonium (NH_4_^+^) aerosol, fine dust, and fine sea salt constitute fine particulate matters that are 2.5 microns or less in diameter (PM_2.5_) [1]. PM_2.5_ can enter the respiratory tract, reach the lungs, trespass through the alveoli blood barrier, and enter the bloodstream, affecting multiple organs and systems in the human body [2].

Short and long-term exposure to ambient PM_2.5_ has been associated with several health impacts, such as mortality and incidence of ischemic heart disease, stroke, respiratory diseases, lung cancer, type 2 diabetes, and preterm birth [3]. The disability-adjusted life years (DALYs) attributable to ambient PM_2.5_ were 118.2 million worldwide in 2019, where the DALYs for females were 47.5 million and the DALYs for males were 70.7 million [3]. As of 2019, long-term exposure to ambient PM_2.5_ accounted for 4.14 million deaths worldwide, becoming the sixth leading cause of global disease burden for all ages after other risk factors like high blood pressure, smoking, and high blood sugar [4]. The global annual average concentration of PM_2.5_ in 2021 exceeded the target in the World Health Organization’s (WHO) Global Air Quality Guidelines (AQG) of 5 μg/m^3^, affecting more than 90% of the world’s population [1]. Ambient PM_2.5_ main impact on global deaths and DALYs are ischemic heart disease, stroke, chronic obstructive pulmonary disease (COPD), diabetes mellitus, and lung cancer [3]. From 1990 to 2019, global deaths attributed to ambient PM_2.5_ increased by 102.3%, and DALYs increased by 67.7% [3].

The region of North Africa (Egypt, Algeria, Libya, Morocco, and Tunisia) is water-scarce and arid, facing severe dust storms and experiencing a higher disease burden from PM_2.5_ compared to other regions [2,4]. It also suffers from the impacts of climate change, with decreases in rainfall, growing seasons, and agricultural production yields [2].

Major sources of ambient PM_2.5_ emissions in North Africa from the year 1970 to 2015 include buildings (38.2%), transport (21.5%), industrial combustion (17.3%), other sectors (12.4%), the power industry (6%), agriculture (4.5%), and waste (0.2%) [2]. The emissions from buildings include stationary and non-industrial combustion [2]. Industrial combustion includes fuel production, involving crude oil, and industrial manufacturing, involving cement [2]. Other sectors produce emissions from industrial processes involving non-metallic minerals, non-ferrous metals, chemicals, and solvents [2,5]. Power industry emissions include electrical and heat-generating plants [2]. Lastly, agricultural combustion emissions include urea fertilization and lime application [2].

Libya has consistently reported annual PM_2.5_ concentrations above the WHO’s Global Air Quality Guidelines from 1990 to 2015 [2]. Like other North African countries, Libya relies heavily on fossil fuels, petroleum, and natural gas. Libya is also facing pressure to embrace rapid development, urbanization, and industrialization, which have contributed to the high level of ambient air pollution in the country [5]. Additionally, dust and sandstorms in Libya have added to the increased atmospheric dust load, increasing ambient air pollution levels [5]. Moreover, although Libya’s public health status has improved in recent years, the country still faces significant health challenges related to non-communicable diseases, which were responsible for 73% of the country’s DALYs in 2019 [6]. Diseases like cancer, cardiovascular disease, and diabetes contributed to 33% of the total DALYs in the country in that year [7]. Yet, there has been no comprehensive assessment of the health impacts of ambient particulate matter in Libya. In this study, we aim to describe the burden of disease attributable to ambient PM_2.5_ at the national level in Libya, by age and sex, from 1990 to 2019. This evidence could help decision-makers and stakeholders identify environmental health priorities and support policies to reduce ambient PM_2.5_ in Libya.

## 2. Methods

To estimate the public health impacts of PM_2.5_ pollution in Libya, our methodology leveraged the comprehensive framework in the Global Burden of Disease (GBD) Study 2019 [8], while tailoring our analysis to Libya’s unique environmental and health landscape. Firstly, satellite-based estimates of the aerosol optical depth were used, providing a broad-scale perspective on particulate matter distribution. Secondly, ground-level PM_2.5_ measurements were integrated, offering localized insights into the pollution levels. Thirdly, chemical transport models were utilized to simulate the movement and chemical transformation of pollutants in the atmosphere. These models were combined using spatial and temporal interpolation techniques, ensuring a comprehensive assessment of PM_2.5_ exposure throughout the study period. These components were synthesized to estimate the exposure to PM_2.5_ across different regions within Libya, providing a nuanced understanding of the pollution levels and their temporal trends from 1990 to 2019.

### 2.1. Global Burden of Disease (GBD) Study 2019

This study obtained estimates from the GBD Study 2019 database for Libya from 1990 to 2019 [8]. A detailed description of the methods and results used in the GBD Study 2019 has been published elsewhere [8]. The GBD Study is a collaborative effort that involves over 5000 researchers. Its primary aim is to measure global, regional, and national population health. To achieve this, the study quantified the burden of 369 diseases and injuries (286 causes of death) and 87 risk factors, between 1990 and 2019, for 204 countries and territories. The GBD Study produces estimates of incidence, prevalence, mortality, years lived with disability (YLDs), years of life lost due to premature mortality (YLLs), DALYs, life expectancy (LE), and healthy life expectancy (HALE). DALYs have two main components, YLLs and YLDs, calculated by adding up the deaths in each age group and multiplying it by the standard LE derived from the lowest observed death rate for any age group in countries with a population greater than 5 million. The YLDs are estimated by multiplying the prevalence counts with the disability weight for a disease or injury. To ensure consistency between all the epidemiologic metrics for most causes, DisMod-MR 2.1, a Bayesian meta-regression tool, was used.

**Figure 1:**
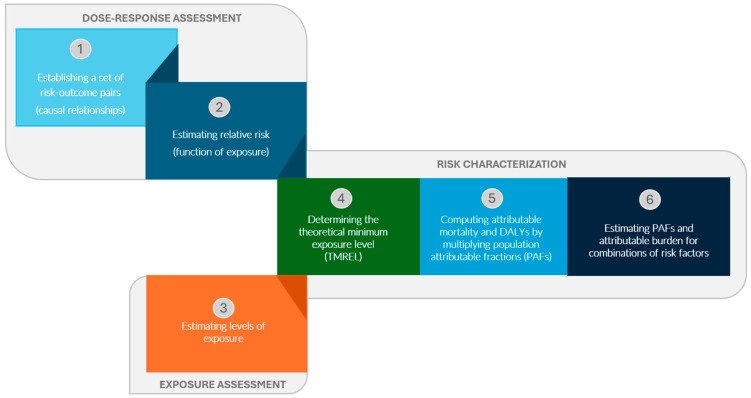
Summary of the comparative risk assessment (CRA) steps (DALYs: disability-adjusted life years).

The GBD Study 2019 identifies attributable mortality and DALYs for 87 risk factors or combinations of risk factors. It follows the general framework established for comparative risk assessments (CRAs), which has been used in the GBD Study since 2002. A CRA comprises six analytical steps (Figure 1): (1) establishing a set of risk–outcome pairs, identified with convincing or probable causal relationships based on the most recent, relevant studies; (2) estimating the relative risks as a function of exposure, based on published systematic reviews and meta-regression analyses; (3) estimating the levels of exposure for each age, sex, location, and year included in the study, based on all available data sources, using methods such as spatiotemporal Gaussian process regression or DisMod-MR 2.1 (a Bayesian meta-regression software); (4) determining the theoretical minimum exposure level (TMREL), a level of exposure associated with the minimum level of risk, based on published trials or cohort studies; (5) computing the attributable mortality and DALYs by multiplying the population attributable fractions (PAFs) with the relevant mortality or DALY estimates for each age, sex, location, and year; and (6) estimating the PAFs and the attributable burden for combinations of risk factors, accounting for the mediation of different risk factors through other risk factors.

This study summarizes the exposure levels for dichotomous, polytomous, and continuous risk factors, using the summary exposure value (SEV) to allow comparisons over time, across locations and risks. The SEV compares the distribution of excess risk times concerning the exposure level to a population, where everyone is at maximum risk, and reflects a measure of exposure to a risk factor normalized on a scale of 0–100, capturing the risk factor’s prevalence and severity or extent. All estimates and their 95% uncertainty intervals (95% UIs) are reported. In addition to mortality, this study reports the DALYs attributable to these risk factors, which facilitates a comparison of fatal and nonfatal health conditions across all diseases and injuries and between different geographic regions. This study’s Supplementary Materials present the ambient particulate matter risk-specific model description. All the presented results are based on the GHDx database, and GBD Compare estimates. To estimate the changes between 1990 and 2019 and allow comparisons between countries, this study mainly relies on age-standardized rates attributable to risk factors and their percent changes since 2010. Furthermore, each SEV age-standardized rate is presented, representing exposure per 100 and 95% uncertainty intervals (UI).

### 2.2. Data Sources and Integration

This study synthesizes PM_2.5_ exposure data for Libya from the GBD Study 2019, employing a comprehensive array of data sources, including satellite-based aerosol optical depth observations, ground-level air quality measurements, and chemical transport models (Table 1). These diverse data sources are integrated using spatial and temporal interpolation techniques to estimate the annual PM_2.5_ exposure levels across Libya from 1990 to 2019.

### 2.3. Exposure Estimation Methodology

The exposure to PM_2.5_ was quantified using a population-weighted annual average concentration across Libya. To compute the annual average population-weighted PM_2.5_ levels, a methodology that integrated spatially resolved PM_2.5_ concentration data with population density information was employed. This approach ensured that the computed averages reflect the exposure levels experienced by the population, rather than merely averaging geographical concentrations. Specifically, the PM_2.5_ concentrations obtained from satellite observations, ground monitoring stations, and chemical transport models were first mapped across the region. These data were then weighted according to population distribution data, sourced from the latest available census and projected estimates, from which the mean PM_2.5_ exposure was calculated. This method accurately represented the inhalation potential of the general population.

**Figure 2:**
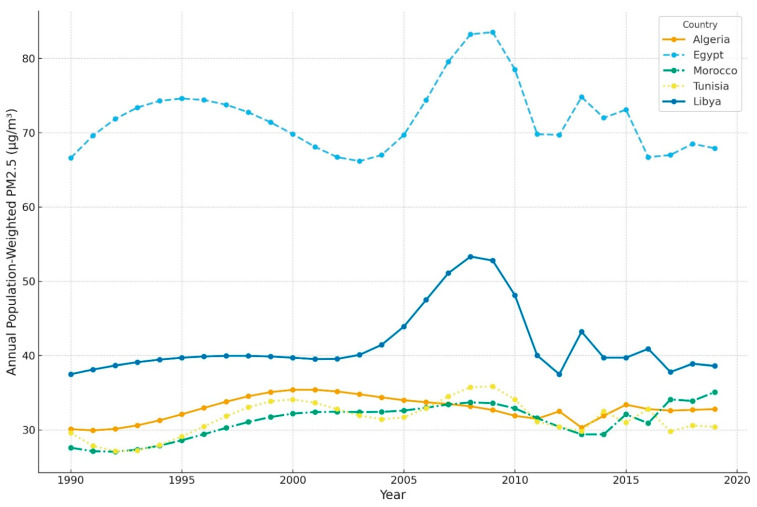
Annual population-weighted mean PM_2.5_ μg/m^3^ levels in Libya and four North African neighboring countries from 1990 to 2019.

The annual average population-weighted PM_2.5_ levels across Libya from 1990 to 2019 are depicted in Figure 2, which also displays a trend in the PM_2.5_ exposure over the three decades and among four North African neighboring countries (obtained using the same exposure assessment approach), highlighting any significant changes or patterns in air quality. Moreover, the figure emphasizes the spatial and temporal distribution of PM_2.5_ and its potential impact on public health over time. Each point or line in the figure corresponds to the computed annual average for a particular year, where color gradients represent the intensity of the PM_2.5_ concentrations. This facilitates an intuitive understanding of changes in air pollution levels and their broader health implications.

The exposure estimation methodology used in this study employed satellite-based aerosol optical depth (AOD) measurements from the moderate-resolution imaging spectroradiometer (MODIS) aboard NASA’s Terra and Aqua satellites. Specifically, the MODIS Collection 6.1 Level 2 aerosol products were employed, which provided the AOD at a wavelength of 550 nanometers, a commonly used wavelength for assessing particulate matter in atmospheric sciences, due to its sensitivity to a wide range of particle sizes. To ensure the accuracy and reliability of these AOD measurements, extensive quality control and quality assurance protocols were implemented. This included using only the highest quality (‘QA_Flag’ of 3) AOD data, which were screened for cloud cover and other atmospheric interferences that could otherwise affect the accuracy of satellite observations. Additionally, the AOD data were validated against ground-based sun photometer measurements from the Aerosol Robotic Network (AERONET). This cross-validation process confirmed the reliability of the satellite-derived AOD values, which were adjusted to align with direct ground-based observations. This rigorous approach to data quality ensured that our AOD measurements were both precise and accurate, providing a solid foundation for subsequent air quality and exposure assessments.

For ground-level PM_2.5_ measurements, data were utilized from diverse sources to ensure comprehensive coverage and accuracy. These measurements were primarily sourced from Global Atmosphere Watch (GAW) stations, supplemented by data from national monitoring networks across various regions. Depending on the data availability from each station or database, the data incorporated into this analysis included daily and hourly observations. This integration of multiple temporal resolutions allowed us to understand the complex dynamics of PM_2.5_ and facilitated a robust calibration of our satellite-derived aerosol optical depth (AOD) measurements against these ground-truth references. The choice of daily and hourly measurements was dictated by the need to accurately capture both short-term fluctuations and longer-term trends in PM_2.5_ concentrations, which are critical for assessing exposure and potential health impacts. In areas where direct PM_2.5_ measurements were unavailable, empirically derived conversion factors were used to estimate the PM_2.5_ concentrations from the available PM_10_ data. These conversion factors were developed based on a comprehensive literature review and validated through comparative studies that analyzed the typical ratio of PM_2.5_ to PM_10_ in similar environmental and meteorological conditions. In regions like Libya, where arid conditions and dust storms are frequent, the conversion factors used to estimate PM_2.5_ concentrations from PM_10_ data were adjusted to reflect the local atmospheric particulate characteristics. Empirical studies specific to similar arid environments suggested using a lower PM_2.5_/PM_10_ ratio, typically ranging from 0.3 to 0.5. This adjustment accounted for the larger proportion of coarse particulate matter, such as dust and sand, in the air, which is prevalent in Libya’s desert climate. Utilizing these tailored conversion factors ensured that our PM_2.5_ estimates were both accurate and representative of the region’s unique environmental conditions.

These datasets were integrated using the Data Integration Model for Air Quality (DIMAQ), which combined satellite measurements with ground-based observations through a chemical transport model, specifically the GEOS-Chem model. Integration was achieved through a statistical matching technique, which aligned and calibrated satellite-derived AOD values with actual PM_2.5_ measurements from ground stations, adjusting for geographical and temporal variations in data availability and quality. This calibration process incorporated a machine learning approach, utilizing regression models to predict PM_2.5_ levels in areas without direct monitoring data.

Quality assurance of these data was conducted through rigorous validation against independent measurement datasets not used in the model calibration process. Regular cross-validation was performed to assess model performance and ensure robustness. These processes ensured that the integrated dataset reflected accurate and reliable PM_2.5_ exposure estimates, forming a credible basis for the health impact assessments presented in this study.

### 2.4. Comparative Risk Assessment (CRA) Framework

This study assessed the health impacts of PM_2.5_ exposure in Libya by adopting the GBD Study 2019’s CRA framework. This included focusing on a select list of health outcomes that were associated with PM_2.5_ exposure, including ischemic heart disease, stroke, chronic obstructive pulmonary disease, lung cancer, and lower respiratory infections. The CRA framework allowed for estimating both the attributable deaths and DALYs due to PM_2.5_, applying country-specific relative risks that reflect the unique epidemiological profile of Libya. Consistent with the GBD Study approach, the TMREL for PM_2.5_ exposure in this study was defined to reflect the lowest observed concentrations associated with health risks based on a review of cohort studies. This critical threshold informed our assessment of the attributable risk and disease burden, enabling focused analysis of the health impacts due to PM_2.5_ levels, exceeding the minimum-risk exposure.

To ensure the reliability and efficacy of our modeling approaches, rigorous statistical methods were employed as a part of the GBD Study’s standardized methodology. This included using DisMod-MR 2.1, a Bayesian meta-regression tool that integrates various data sources, including epidemiological and health data, to estimate global disease incidence, prevalence, and mortality rates. Furthermore, the CRA framework utilized in this study involved a statistical comparison between the model outputs and actual ground-level measurements. This was achieved by calibrating our chemical transport models and satellite-based estimates against empirical data collected from air quality monitoring stations. Additionally, sensitivity analyses were conducted in this study to assess the robustness of our findings and to adjust for potential biases and uncertainties that are inherent in the modeling process. These steps ensured that our models provided a credible and scientifically robust basis for estimating the health impacts of PM_2.5_ exposure, thereby directly addressing concerns about the efficacy of the modeling techniques.

**Figure 3:**
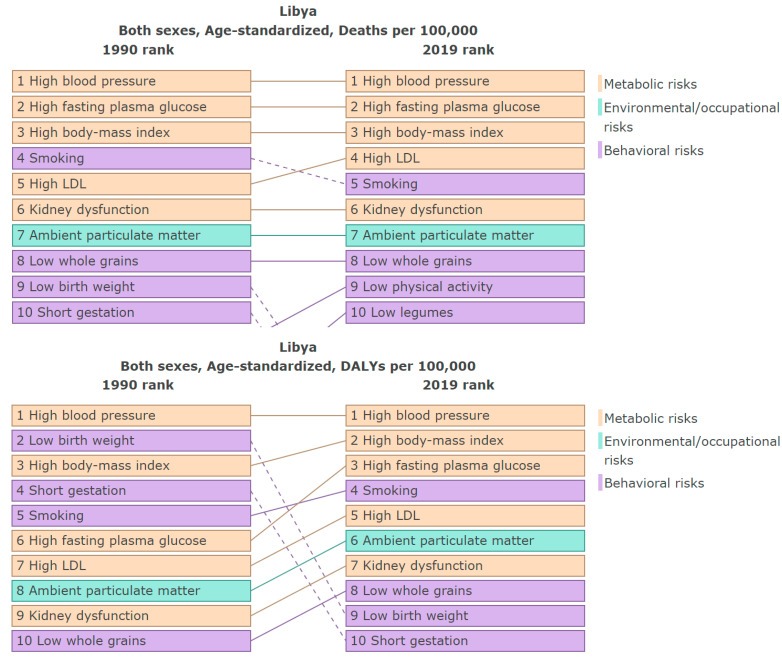
Top 10 causes of death and disability-adjusted life years (DALYs) in Libya, age-standardized, for both sexes (extracted from GBD Compare website [6]).

**Figure 4:**
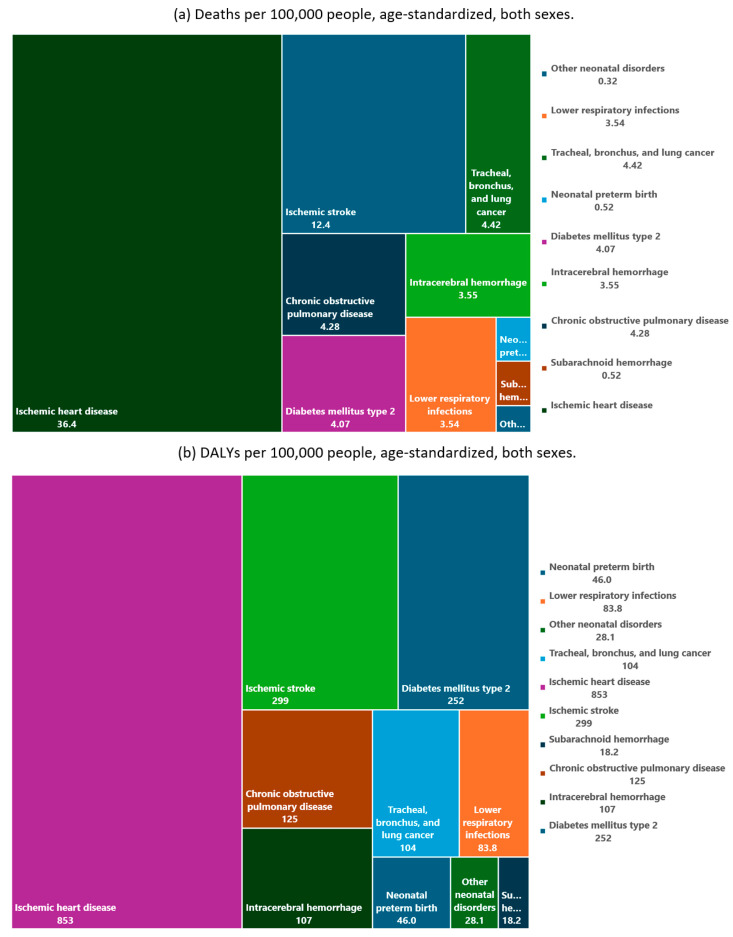
Treemap of (**a**) cause-specific mortality and (**b**) disability-adjusted life years (DALYs) attributed to PM_2.5_ in Libya, for 2019, per 100,000 people (both sexes, age-standardized).

While our methodology is grounded in the established GBD Study framework, our study distinguishes itself through its focused examination of PM_2.5_ pollution and the associated health implications in Libya. By analyzing the data on the global burden of disease from a local perspective, we can gain a better understanding of the epidemiological trends specific to Libya. This targeted analysis not only addresses a significant gap in the literature, but also provides a resource for policymakers and public health officials within Libya to implement targeted interventions to mitigate the health impacts of air pollution. The data presented in Figure 3 are derived from the analysis of the GBD Study 2019, specifically from the CRAs in 1990 and 2019, where metabolic, environmental, occupational, and behavioral risk factors are ranked using the death rates and DALY rates per 100,000 people [6]. Table 2 and Figure 4 present the absolute (Table 2) and relative values using rates per 100,000 (Figure 4), quantifying the attributable deaths, DALYs, YLLs, and YLDs across various diagnoses attributable to PM_2.5_ in Libya.

## 3. Results

In 2019, the average exposure to ambient particulate matter in Libya was 38.6 μg/m^3^ (95% UI 26.1–54.9), as shown in Figure 2. Over the past three decades (from 1990 to 2019), there has been a 3% rise (1.1 μg/m^3^) in the average level of particulate matter in the air in Libya. This type of pollution was the seventh leading cause of mortality in Libya in 1990 and continued to have a significant impact until 2019, as seen in Figure 3. In 2019, 3368 deaths (95% UI 2381–4613) were attributed to particulate matter (Table 2). Particulate matter was responsible for 11% of all deaths that occurred in Libya in 2019. Older adults, over the age of 70, accounted for the majority of these deaths, approximately 67% (5897 deaths per 100,000).

In terms of cause-specific absolute mortality for both sexes, ischemic heart disease (IHD) accounted for 53% (1799) of all deaths attributable to PM_2.5_ followed by ischemic stroke with 17% (559); total cancers 6% (215); tracheal, bronchus, and lung cancer 6% (215); intracerebral hemorrhage 6% (197); type 2 diabetes 5% (185); chronic obstructive pulmonary disease (COPD) 5% (184); lower respiratory infections (LRI) 5% (159); and neonatal preterm birth 1% (20) (Table 2).

In absolute numbers, deaths attributable to PM_2.5_ almost tripled in 2019 (3368 (95% UI 2381–4613)), compared to 1990 (1303 (95% UI 854–1775)) for all ages. The age-adjusted mortality rate attributable to PM_2.5_ increased by 13% in 2019 (70 (95% UI 50–96)), compared to 1990 (62 (95% UI 40–85)) (Table S1).

There were differences in the cause-specific, age-adjusted PM_2.5_ mortality between males and females in 2019. In 2019, ischemic heart disease was the primary cause of cause-specific mortality among males, followed by ischemic stroke; tracheal, bronchus, and lung cancer; total cancers; chronic obstructive pulmonary disease; lower respiratory infections; type 2 diabetes; intracerebral hemorrhage; neonatal preterm birth; subarachnoid hemorrhage; and other neonatal disorders (Figure S4). For females, ischemic heart disease and ischemic stroke were the primary causes of cause-specific mortality. In contrast, type 2 diabetes emerged as the next driver, followed by lower respiratory infections; chronic obstructive pulmonary disease; total cancers; tracheal, bronchus, and lung cancer; subarachnoid hemorrhage; neonatal preterm birth; and other neonatal disorders (Figure S5).

Among all ages, 107,207 (95% UI 76,991–142,992) of the total DALYs were attributed to PM_2.5_ in Libya in 2019 (Table 2). From these, 77% (214,258 DALYs per 100,000) were in people younger than 70 years old, and 23% (62,867 DALYs per 100,000) were in people older than 70 years old, in 2019 (Figure S1). The age-adjusted DALY rates attributable to PM_2.5_ increased by 8.5% in 2019 (1779, (95% UI 1170–2442)) compared to 1990 (1930 (95% UI 1402–2546)) (Table S1). The sum of the YLLs and YLDs together constitute the DALYs. In 2019, YLLs represented 83% of the DALYs, and YLDs represented 17% of the DALYs (Table 2). In 2019, YLD was driving the PM_2.5_ health burden, where 89,113 (95% UI: 63,554–121,967) YLLs and 18,094 (95% UI: 11,694–25,068) YLDs were attributed to PM_2.5_ (Table 2). From the total DALYs, ischemic heart disease 46% (49,307) and ischemic stroke 15% (15,871) contributed the most, followed by type 2 diabetes 13% (13,755), intracerebral hemorrhage 7% (7008), COPD 6% (6555), total cancers 5% (5672), tracheal, bronchus, and lung cancer 5% (5672), LRI 4% (4352), and neonatal pre-term birth 2% (1805), in 2019, for both sexes (Table 2). In 1990, PM_2.5_ was Libya’s eighth leading cause of DALYs (age-standardized rates). However, its impact increased, becoming the sixth leading cause of DALYs in 2019 (Figure 3). In relative numbers, using age-standardized rates per 100,000 in both sexes (Figure 4), ischemic heart disease was the most important cause of death related to PM_2.5_ in Libya, followed by ischemic stroke, and the same pattern was shown in terms of DALYs age-standardized rates per 100,000 in both sexes.

## 4. Discussion

The present study represents the most comprehensive assessment of Libya’s disease burden associated with ambient particulate matter (PM_2.5_). Our findings indicate that long-term exposure to ambient PM_2.5_ in Libya resulted in 3368 deaths and 107,207 lost years of healthy life in 2019. Specifically, PM_2.5_ was responsible for 11% of all deaths in the country and was ranked as the seventh leading health risk factor (Figure 3). Notably, while rates of PM_2.5_-related mortality and cardiovascular mortality decreased globally in high-income nations from 1990 to 2019 due to improvements in air quality, such rates have increased in Libya. This trend may be partly attributed to rising air pollution levels and the absolute number of deaths. Indeed, compared to 1990, Libya experienced a 13% increase in mortality rates and an 8.5% increase in DALY rates in 2019 (Figures S2 and S3).

Compared to other North African nations, Libya ranked fourth in terms of mortality rates attributable to PM_2.5_ in 2019, reporting 70.1 age-standardized deaths per 100,000 individuals. Egypt, Morocco, and Algeria were ahead of Libya, with 157.5, 97.4, and 76.5 age-standardized deaths per 100,000 people, respectively (Figure S2).

Regarding DALYs, Libya reported the third highest rate in 2019, with 1930 age-standardized DALYs per 100,000 individuals. Egypt and Morocco had higher rates of 3993 and 2426 age-standardized DALYs per 100,000 individuals, respectively (Figure S3). Nevertheless, in Libya, between 2009 and 2019, all-cause mortality rates and DALY rates attributable to PM_2.5_ decreased by 18% and 11%, respectively (age-standardized for both sexes) (Figures S2 and S3). This decrease could be associated with investments in health and improvements in health outcomes implemented by the national government [9]. In contrast to other North African countries, Libya has reported a steady but less variable PM_2.5_ attributable DALY rate since 1990. However, its DALY rate has not decreased, unlike in Egypt, Algeria, and Tunisia (Figure S3).

One of the contributing factors to the lack of improvement in the DALY rates in Libya could be the political instability. Another contributing factor is the country’s increased development and reliance on fossil fuel-fired thermal power plants and crude oil production, since the 1980s [10,11]. Oil is responsible for 40 percent of the country’s total economic output and 95 percent of its export earnings [11]. The political situation has also put strain on the healthcare system, leading to a rise in the incidence of infectious and non-infectious diseases [12,13]. As a result, access to medical care could be limited, leading to increased YLLs and DALYs. Compared to Morocco, Algeria, Tunisia, and Egypt, Libya has been at increased risk of drought since 1990. Droughts, in combination with increased seasonal heat (May-October) and Saharan dust re-suspension, could also contribute to the steady air pollution DALY rates, with ischemic heart disease, stroke, lung cancer, and preterm births as the primary drivers [13].

After adjusting for population size, it was observed that the health burden associated with PM_2.5_-related mortality and DALYs was higher among males and those aged 70 years or older (Figures S1, S4 and S5). This trend is particularly noticeable in middle-income countries such as Libya, where the population is aging. Males were responsible for contributing to 56% (60,497 (95% UI 82,107–42,920)) of DALYs, while females contributed to 44% (46,710 (95% UI 60,811–34,099) of DALYs. The impact of air pollution varied between men and women due to sex-linked biological differences, including hormonal complement, body size, and gender-specific differences in co-exposures, such as occupational exposure, lifestyle factors, and activity patterns [14]. Furthermore, the third leading cause of PM_2.5_-attributable DALYs and mortality for females in 2019 was type 2 diabetes (Figures S4 and S5). Epidemiological studies have shown that long-term exposure to PM_2.5_ leads to insulin resistance through oxidative damage, endothelial dysfunction, and inflammation [15]. Additionally, PM_2.5_ can alter immune responses in visceral adipose tissue and induce endoplasmic reticulum stress, leading to changes in insulin transduction, insulin sensitivity, and glucose metabolism [15,16].

The population-weighted PM_2.5_ concentrations for Libya have increased steadily over the past 29 years, rising from 37.5 μg/m^3^ in 1990 to 38.6 μg/m^3^ in 2019, with a peak of 48.1 μg/m^3^ in 2010 (Figure 2). These levels have consistently exceeded the recommended exposure level set by the WHO AQG in 2021 of 5 μg/m^3^ [17]. Although there have been variations in PM_2.5_ concentrations in the region between 2010 and 2019, with a notable reduction in 2012 and an increase in 2013 (Figure 2), these fluctuations may be attributed to weather-related dust and sandstorms, and similar patterns can be shown in North African neighboring countries. For instance, in May 2017, Libya experienced its heaviest recorded dust storm, which led to a heavy dust load, impaired visibility, cloud formation, and a decline in air quality, negatively affecting all sectors, including transportation, telecommunications, infrastructure, electricity, and intensifying drought effects [5]. Particulate matter comprises fine or coarse particles, derived from natural or anthropogenic sources. Fine particles originate from trace components of anthropogenic origin, such as elemental carbon and sulfur, while coarse particles originate from naturally derived trace metals, such as aluminum and silicon [18,19]. During seasonal dust storms, coarse particles, mainly composed of dust, constitute a larger fraction of the total particulate matter less than 10 μm in diameter (PM_10_) [20]. However, in Libya, most of the PM_2.5_ emissions come from anthropogenic activities, such as electrical power plants, transportation, residential and commercial sectors with diesel and gasoline-fueled cars and electricity generators, cement manufacturing factories, lead melting, solid waste incineration, quarries, and crude oil refinery industries [5].

The energy sector in Libya relies heavily on crude oil, which is the major energy source [21]. In 2010, Libya was ranked as the 31st largest producer of crude oil globally, with a daily production rate of 2 billion barrels [21]. By early 2016, the country had produced a total of 48.4 billion barrels [21]. However, the process of extracting, refining, transmitting, and burning crude oil results in ambient air pollution [21]. The combustion of crude oil leads to corrosion and air pollution due to its high sulfur content, which can react with oxygen to form sulfur dioxide, a naturally occurring gas that harms human health and the environment [22,23]. The demand for power generation and other products from refined oil, driven by the rapid urbanization in Libya, has further intensified air pollution from burning crude oil [24]. This has contributed to an increase in PM_2.5_ concentrations in the atmosphere, further exacerbated by the effects of industrial pollutant emissions, coal smoke emissions, open garbage incineration, and exhaust emissions from old cars [25]. Despite being a signatory to the United Nations Framework Convention on Climate Change (UNFCCC) and the Paris Agreement on Climate Change (2015), Libya often lacks adequate regulations or systems to monitor and control air pollution [21,26]. Evidence has suggested that existing policies in the transport, energy, waste management, and industry sectors could exacerbate the impact of anthropogenic sources of air pollution [26]. Therefore, policies aimed at reducing anthropogenic sources of air pollution and improving energy efficiency by shifting to sustainable, renewable energies would be instrumental in promoting air quality.

This study examines the impact of ambient PM_2.5_ exposure and its effects on long-term health, including mortality rates due to air pollution. In Libya, anthropogenic sources are the main culprits of PM_2.5_, making it a more important factor to assess than PM_10_. Additionally, this study assumes that all particles, regardless of origin or makeup, have the same level of toxicity per unit mass. This assumption is supported by the WHO and the United States Environmental Protection Agency (EPA) [27]. While previous research suggested that PM_2.5_ toxicity could vary based on its combustion source, there is no evidence to identify specific sources or components that determine PM_2.5_ mixture toxicity [27,28]. Therefore, current evidence does not support using source-specific relative risk functions to estimate burdens, leading to uncertainty in our estimates for this study.

The preceding analysis highlighted that air pollution is a primary health risk factor in Libya, where PM_2.5_ contributes to non-communicable diseases. Previous studies in other countries have similarly reported air pollution as a significant health risk factor, with metabolic risk factors such as high blood pressure, high body-mass index, and high fasting plasma glucose identified as primary drivers [9,28,29,30]. The top 10 causes of PM_2.5_-attributable deaths and DALYs in Libya were also found to be non-communicable diseases, consistent with other countries, such as Egypt and Saudi Arabia [28]. Furthermore, the population-weighted PM_2.5_ exposure in Libya is above the WHO AQG target (38.6 μg/m^3^ in 2019) and close to the global exposure level of 42.6 μg/m^3^.

The present study possesses several noteworthy strengths, including being the first in Libya to analyze the ambient particulate matter burden of disease between 1990 and 2019. The study employed rigorous methodologies to develop a comprehensive health analysis, and all-cause mortality was analyzed. Although harmonized, comparable data and analysis were utilized among countries, the lack of subnational data for Libya poses a significant challenge for policymakers in identifying local sources of air pollution and their health impacts. Additionally, the absence of daily, monthly, and seasonal air pollution measurements in this study fails to capture the short-term impacts of PM_2.5_, such as seasonal and temporal changes like heatwaves, droughts, sandstorms, and dust storms. While this study only focused on assessing the long-term impacts of air pollution using annual average concentrations, which captured most of the health burden associated with PM_2.5_, it is essential to conduct seasonal analyses to describe the short-term impacts of air pollution in the region, including the effects of weather and climate-related events. However, it is important to note that the GBD Study has several limitations, including data availability and uncertainty, coding practices, and limitations in the current analytical tools. Differences in political conflicts, social and cultural factors, and access to natural and economic resources may have influenced the comparison of diseases and disability burdens between Libya and its neighboring countries in the Middle East and North Africa (MENA) region. Moreover, this study may have underestimated the disease burden attributable to PM_2.5_, due to a lack of assessments of other potential PM_2.5_-related health outcomes. Systematic reviews have suggested a correlation between PM_2.5_ exposure and kidney diseases, low-birth weight and preterm birth, and asthma in children and adults, but due to the high heterogeneity among studies, those outcomes were not included in these analyses [31,32,33]. Additionally, ambient air pollutants such as ozone and nitrogen dioxide and other sources of exposure, such as household air pollution, were not assessed, which could have generated multiple health impacts. Therefore, this study may have underestimated the overall health impact of ambient air pollution, despite being the primary source of air pollution exposure in Libya, compared to household sources.

## 5. Conclusions

In conclusion, this study comprehensively assesses the burden of disease associated with ambient particulate matter (PM_2.5_) in Libya. The findings indicate that long-term exposure to ambient PM_2.5_ is responsible for a significant proportion of deaths and lost years of healthy life in the country. PM_2.5_ is ranked as the seventh leading risk factor for health, and the situation has worsened over the years, with an increase in mortality and DALY rates. Furthermore, this study shows that the population-weighted PM_2.5_ concentrations have steadily increased over the years, exceeding the recommended exposure levels set by the WHO AQG. Therefore, the findings underscore the urgent need for measures to reduce PM_2.5_ pollution in Libya and other North African countries to improve the health outcomes of their populations.

## Figures and Tables

**Table 1:** Summary of the main parameters used in the analysis.

Dataset	Parameter	Description	Usage in Study
MODIS (Terra and Aqua)	Moderate-resolution imaging spectroradiometer (MODIS). Collection 6.1 multi-angle implementation of atmospheric correction (MAIAC) aerosol optical depth (AOD) at 550 nm.	This dataset provides daily global aerosol optical depth measurements using the MAIAC algorithm.	Used to estimate ground-level PM_2.5_ concentrations. The high-resolution AOD data were correlated with ground-based measurements to model PM_2.5_ across various geographical areas.
Ground Monitoring Stations	Fine particulate matters that are 2.5 microns or less in diameter (PM_2.5_). Daily average concentrations.	Direct measurements from air quality monitoring stations worldwide. This includes both urban and rural stations providing 24 h mean concentrations.	These measurements are the primary validation and calibration source for our satellite data estimates and chemical transport model outputs.
GEOS-Chem Model	PM_2.5_ composition simulation.	The model simulates global atmospheric chemistry and output concentrations of particulate components, such as sulfate, nitrate, ammonium, organic carbon, and black carbon.	We use these simulated outputs to provide background levels of PM_2.5_ and to understand how chemical composition affects health outcomes in different regions.
Population Data (GPW, v4)	Population count per grid cell.	High-resolution population count grids that estimate human population counts based on census data, adjusted for undercounting and overcounting, as well as for births, deaths, and migration.	Used to calculate population-weighted PM_2.5_ exposure. This data helps assess the exposure levels more accurately by factoring in the population density across different regions.

**Table 2:** Libya burden of disease related to fine particulate matter smaller than 2.5 μm (PM_2.5_), 1990, 2000, 2010, and 2019, by diagnosis, in absolute number, all ages and both sexes.

	Year	1990				2019			
	Measure	Deaths	DALYs	YLLs	YLDs	Deaths	DALYs	YLLs	YLDs
All causes	Value (Lower Upper)	1303 (854–1775)	54,767 (35,125–75,102)	51,520 (32,958–71,442)	3248 (1944–4725)	3368 (2381–4613)	107,207 (76,991–142,992)	89,113 (63,554–121,967)	18,094 (11,694–25,068)
Ischemic heart disease	Value (Lower Upper)	541 (336–776)	14,656 (9290–21,110)	14,383 (9135–20,788)	274 (152–437)	1799 (1264–2566)	49,307 (34,729–71,024)	48,150 (33,891–69,681)	1157 (724–1752)
Ischemic Stroke	Value (Lower Upper)	133 (81–204)	3347 (2100–4979)	2640 (1617–4015)	707 (412–1039)	559 (362–826)	15,871 (10,830–22,145)	12,453 (8036–18,021)	3418 (2303–4705)
Type 2 Diabetes	Value (Lower Upper)	37 (20–55)	2052 (1160–2969)	812 (454–1205)	1240 (642–1976)	185 (122–271)	13,755 (8816–19,596)	4204 (2704–6213)	9551 (5551–14,465)
Intracerebral hemorrhage	Value (Lower Upper)	101 (61–149)	3248 (1979–4781)	3048 (1832–4529)	200 (116–300)	197 (123–295)	7008 (4431–10,102)	6399 (3914–9473)	609 (397–851)
Chronic obstructive pulmonary disease	Value (Lower Upper)	59 (35–88)	1771 (1147–2489)	1116 (675–1667)	655 (424–946)	184 (107–275)	6555 (4079–9501)	3584 (2063–5380)	2970 (1871–4168)
Tracheal, bronchus, lung cancers	Value (Lower Upper)	67 (37–99)	1743 (986–2603	1728 (978–2588)	15 (8–25)	215 (130–311)	5672 (3420–8230)	5624 (3392–8166)	48 (27–77)
Lower respiratory infections	Value (Lower Upper)	137 (79–212)	8528 (4614–14,019)	8456 (4572–13,932)	72 (37–126)	159 (89–256)	4352 (2399–7077)	4267 (2359–6969)	85 (42–149)
Neonatal preterm birth	Value (Lower Upper)	135 (56–227)	12,032 (5013–20,160)	12,026 (5010–20,153)	5 (3–8)	20 (8–35)	1805 (681–3148)	1802 (679–3145)	1176 (745–1659)
Other neonatal disorders	Value (Lower Upper)	44 (18–78)	3924 (1604–6919)	3922 (1602–6914)	2 (1–3)	12 (4–24)	1101 (322–2144)	1101 (322–2143)	1 (0–1)
Neonatal encephalopathy due to birth asphyxia and trauma	Value (Lower Upper)	21 (8–41)	1861 (673–3667)	1861 (673–3667)	1 (0–1)	3 (1–6)	270 (89–516)	269 (89–516)	1 (0–1)
Subarachnoid hemorrhage	Value (Lower Upper)	17 (9–27)	618 (360–959)	551 (309–884)	67 (38–104)	30 (18–42)	1272 (789–1996)	1024 (586–1726)	247 (154–361)
Neonatal sepsis and other neonatal infections	Value (Lower Upper)	5 (2–9)	407 (162–803)	407 (161–803)	0 (0–0)	2 (1–4)	176 (69–351)	176 (69–351)	0 (0–0)
Diarrheal diseases	Value (Lower Upper)	3 (1–6)	236 (81–576)	231 (78–568)	5 (2–9)	0 (0–1)	26 (10–50)	22 (8–45)	4 (1–7)
Hemolytic diseases and other neonatal jaundice	Value (Lower Upper)	2 (1–4)	226 (91–427)	221 (88–420)	5 (2–9)	0 (0–0)	19 (8–36)	19 (8–35)	0 (0–0)
Meningitis	Value (Lower Upper)	1 (0–2)	72 (28–138)	72 (28–138)	0 (0–0)	0 (0–0)	6 (2–11)	5 (2–11)	0 (0–0)
Upper respiratory infections	Value (Lower Upper)	0 (0–0)	1 (0–2)	0 (0–0)	1 (0–2)	0 (0–0)	1 (0–1)	0 (0–0)	1 (0–1)
Sudden infant death syndrome	Value (Lower Upper)	0 (0–1)	41 (8–127)	41 (8–127)	N/A	0 (0–0)	9 (2–19)	9 (2–19)	N/A
Otitis media	Value (Lower Upper)	0 (0–0)	0 (0–0)	0 (0–0)	0 (0–0)	0 (0–0)	0 (0–0)	0 (0–0)	0 (0–0)
Encephalitis	Value (Lower Upper)	0 (0–0)	5 (2–10)	5 (2–10)	0 (0–0)	0 (0–0)	3 (1–5)	3 (1–5)	0 (0–0)

## Data Availability

All the data and analyses used in this study were obtained from the GBD Study 2019, which is publicly available online on the website of the Institute of Health Metrics and Evaluation (https://vizhub.healthdata.org/gbd-compare/ accessed on 18 May 2024).

## Supplementary Materials

The following supporting information can be downloaded at: https://www.mdpi.com/article/10.3390/ijerph21060667/s1. Table S1. Libya burden of disease related to fine particulate matter smaller than 2.5 μm (PM 2.5), 1990, 2000, 2010, and 2019, by year, and diagnosis, rate per 100,000, age-standardized; Figure S1. Deaths (a) and Disability-adjusted life years, DALYs (b) attributable to PM2.5 Libya in 2019, by age and both sexes (per 100,000); Figure S2. Annual deaths (all-cause) attributable to fine particulate matter smaller than 2.5 μm (PM2.5) per 100,000 people (both sexes, ages-standardized); Figure S3. Disability-adjusted life years (DALYs) attributable to fine particulate matter smaller than 2.5 μm (PM2.5) per 100,000 people (both sexes, ages-standardized); Figure S4. Treemap of (a) cause-specific mortality and (b) disability-adjusted life years (DALYs) attributed to PM2.5 in Libya, 2019, per 100,000 people (males, age-standardized); Figure S5. Treemap of (a) cause-specific mortality and (b) disability-adjusted life years (DALYs) attributed to PM2.5 in Libya, 2019, per 100,000 people (females, age-standardized).
