# Supplementary material for: Public Health Impacts of Ambient Particulate Matter Pollution in Libya from 1990 to 2019: An Analysis of the 2019 Global Burden of Disease (GBD) Study
Source: Int J Environ Res Public Health. 2024 May 23;21(6):667. doi: 10.3390/ijerph21060667 (PMC11204245; doi:10.3390/ijerph21060667)
Supplement: Supplementary file 1 [file ijerph-21-00667-s001.zip › ijerph-2926478-supplementary.pdf]

Supplemental material

Public health impacts of ambient particulate matter pollution in Libya from 1990 to 2019: an analysis form the 2019 Global Burden of Disease study

D. Rojas-Rueda [a,b] *, S. Lamsal [a,b], Mohini Kak [c], Sameh El Saharty [c], Christopher H. Herbst [c]

[a] *Department of Environmental and Radiological Health Sciences, Colorado State University, Fort Collins, CO, USA*

[b] *Colorado School of Public Health, Colorado State University, Fort Collins, CO, USA*

[c] *Health, Nutrition and Population Global Practice, The World Bank, Washington DC, USA*

- The findings, interpretations, and conclusions expressed in this work are those of the authors, and do not necessarily reflect the views of the World Bank or its Board of Directors.

*Corresponding author. Colorado State University, Environmental Health Building, 1601 Campus Delivery, 80523, Fort Collins, USA. *E-mail address:* David.Rojas@colostate.edu (D. Rojas-Rueda).

Contents

Supplemental material.. 1

Public health impacts of ambient particulate matter pollution in Libya from 1990 to 2019: an analysis form the 2019 Global Burden of Disease study 1

METHODS - Ambient particulate matter pollution 3

Exposure Definition. 3

Input data. 4

$PM_{2.5}$ ground measurement database. 4

Satellite-based estimates. 4

Population data. 5

Chemical transport model simulations. 5

Theoretical minimum-risk exposure level. 5

Relative risks and population attributable fractions. 5

RESULTS 6

Table S1. Libya burden of disease related to fine particulate matter smaller than 2.5um (PM 2.5), 1990, 2000, 2010, and 2019, by year, and diagnosis, rate per 100,000, age-standardized. 6

Figure S1. Deaths (a) and Disability-adjusted life years, DALYs (b) attributable to PM2.5 Libya in 2019, by age and both sexes (per 100,000). 8

Figure S2. Annual deaths (all-cause) attributable to fine particulate matter smaller than 2.5µm (PM2.5) per 100,000 people (both sexes, ages-standardized). 9

Figure S3. Disability-adjusted life years (DALYs) attributable to fine particulate matter smaller than 2.5µm (PM2.5) per 100,000 people (both sexes, ages-standardized). 10

Figure S4. Treemap of (a) cause-specific mortality and (b) disability-adjusted life years (DALYs) attributed to PM2.5 in Libya, 2019, per 100,000 people (males, age-standardized). 11

Figure S5. Treemap of (a) cause-specific mortality and (b) disability-adjusted life years (DALYs) attributed to PM2.5 in Libya, 2019, per 100,000 people (females, age-standardized). 13

METHODS - Ambient particulate matter pollution

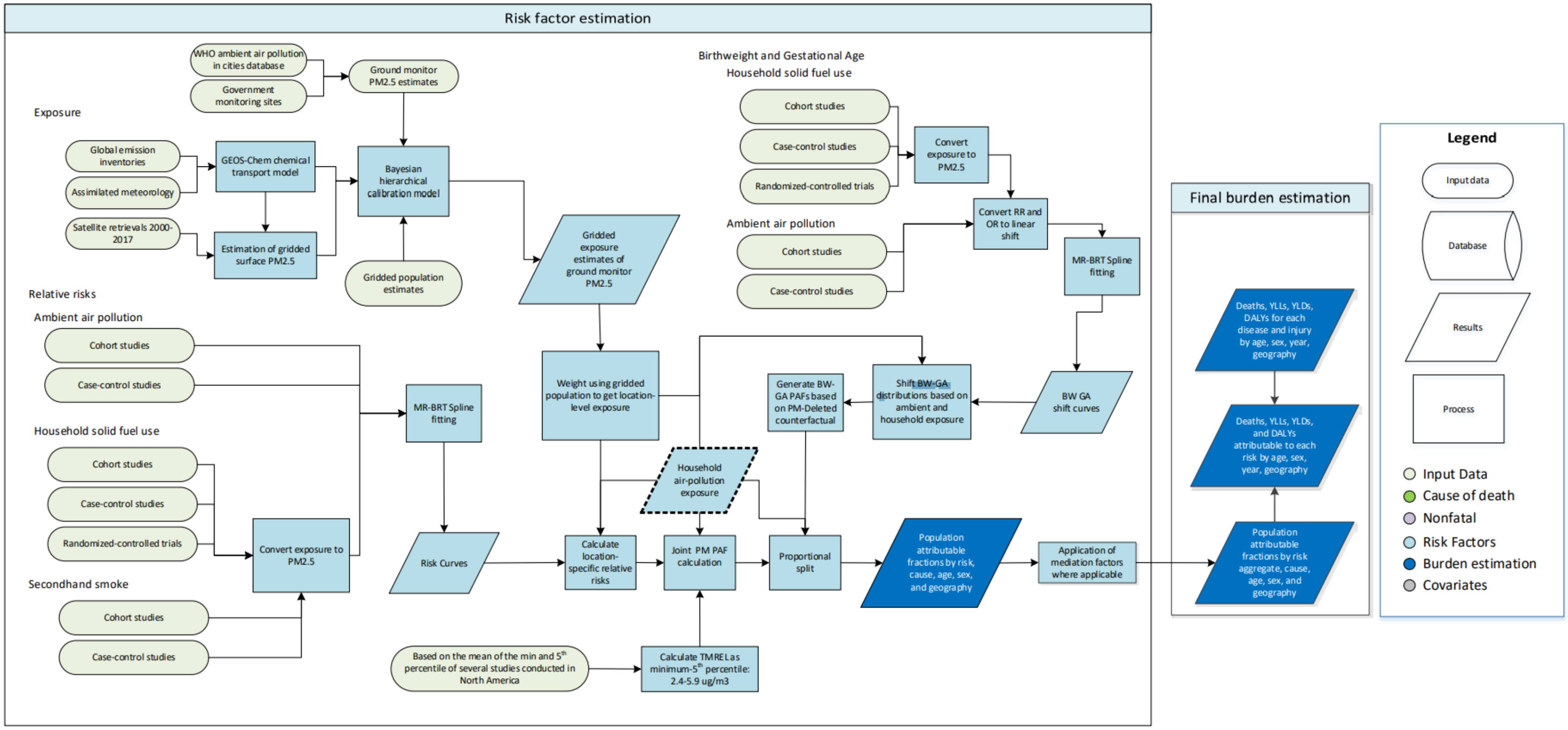

Exposure Definition.

Exposure to ambient particulate matter pollution is defined as the population-weighted annual average mass concentration of particles with an aerodynamic diameter of less than 2.5 micrometers (PM2.5) in a cubic meter of air. This measurement is reported in µg/m3.

Input data.

The data used to estimate exposure to ambient particulate matter pollution comes from multiple sources, including satellite observations of aerosols in the atmosphere, ground measurements, chemical transport model simulations, population estimates, and land-use data.

The following details the updates in methodology and input data used in GBD 2019.

$PM_{2.5}$ ground measurement database.

Ground measurements used for GBD 2019 include updated measurements from sites included in 2017 and additional measurements from new locations. New and up-to-date data (mainly from the USA, Canada, EU, Bangladesh, China, and USA embassies and consulates) were added to the data 78 from the 2018 update of the WHO Global Ambient Air Quality Database used in GBD 2017. The updated data included measurements of concentrations of PM_{10} and $PM_{2.5}$ from 10,408 ground monitors from 116 countries from 2010 to 2017. Most measurements were recorded in 2016 and 2017 (as there is a lag in reporting measurements, few data from 2018 or newer were available). Annual averages were excluded if they were based on less than 75% coverage within a year. If the coverage information was unavailable, then data were included unless sufficient data was already within the same country (monitor density greater than 0.1). For locations measuring only PM_{10}, $PM_{2.5}$ measurements were estimated from PM_{10}. This was performed using a hierarchy of conversion factors ($PM_{2.5}$/PM_{10} ratios): (i) for any location, a 'local' conversation factor was used, constructed as the ratio of the average measurements (of $PM_{2.5}$ and PM10) from within 50km of the location of the PM_{10} measurement, and within the same country, if such measurements were available; (ii) if there was not sufficient local information to construct a conversion factor then a country-wide conversion factor was used; and (iii) if there was no appropriate information within a country, then a regional factor was used. In each case, to avoid the possible effects of outliers in the measured data (both $PM_{2.5}$ and PM_{10}), extreme values of the ratios were excluded (defined as being greater/lesser than the 95% and 5% quantiles of the empirical distributions of conversion factors). As with GBD 2013, 2015, 2016, and 2017 databases, in addition to values of $PM_{2.5}$ and whether they were direct measurements or converted from PM10, the database also included additional information, where available, related to the ground measurements such as monitor geo-coordinates and monitor site type.

Satellite-based estimates.

The global geophysical $PM_{2.5}$ estimates for 2000–2017 are from Hammer and colleagues' Version V4.GL.03.NoGWR used at 0.1o x0.1o resolution (~11 x 11 km resolution at the equator). 1 The method is based on the algorithms of van Donkelaar and colleagues (2016) as used in GBD 2017, 2 with updated satellite retrievals, chemical transport modeling, and ground-based monitoring. The algorithm uses aerosol optical depth (AOD) from several updated satellite products (MAIAC, MODIS C6.1, and MISR v23), including finer resolution, increased global coverage, and improved long-term stability. Ground-based observations from a global sunphotometer network (AERONET version 3) combine different AOD information sources. This is the first time that data from MAIAC at 1 km resolution was used to estimate PM2.5 at the global scale. The GEOS-Chem chemical transport model with updated algorithms was used for geophysical relationships between surface PM2.5 and AOD. Updates to the GEOS-Chem simulation included improved mineral dust and secondary organic aerosol representation and updated emission inventories. The resultant geophysical PM2.5 estimates are highly consistent with ground monitors worldwide (R2 =0.81, slope = 1.03, n = 2541).

Population data.

A comprehensive set of population data, adjusted to match the UN2015 Population Prospectus, on a high-resolution grid was obtained from the Gridded Population of the World (GPW) database. Estimates for 2000, 2005, 2010, 2015, and 2020 were available from GPW version 4, with estimates for 1990 and 1995 obtained from GPW version 3. These data are provided on a 0.0083o × 0.0083o resolution. Aggregation to each 0.1o ×0.1o grid cell was accomplished by summing the central 12 × 12 population cells. Population estimates for 2001–2004, 2006–2009, 2011–2014, and 2016–2019 were obtained by interpolation using natural splines with knots placed in 2000, 2005, 2010, 2015, and 2020. This was performed for each grid cell.

Chemical transport model simulations.

Estimates of the sum of particulate sulfate, nitrate, ammonium, and organic carbon and the compositional concentrations of mineral dust simulated using the GEOS Chem chemical transport model and a measure combining elevation and the distance to the nearest urban land surface (as described in van Donkelaar and colleagues 20162 and Hammer and colleagues (submitted)) 1 were available for 2000–2017 for each 0.1o ×0.1o grid cell.

Theoretical minimum-risk exposure level.

The TMREL was defined as a uniform distribution rather than a fixed value to represent the uncertainty regarding the level at which the scientific evidence was consistent with the adverse effects of exposure. The TMREL was assigned a uniform distribution with lower/upper bounds given by the average of the minimum and fifth percentiles of outdoor air pollution cohort studies exposure distributions conducted in North America, with the assumption that current evidence was insufficient to precisely characterize the shape of the concentration-response function below the fifth percentile of the exposure distributions. The specific outdoor air pollution cohort studies selected for this averaging were based on the criteria that their fifth percentiles were less than that of the American Cancer Society Cancer Prevention II (CPSII) cohort's fifth percentile of 8.2 based on Turner and colleagues (2016).10 This criterion was selected since GBD 2010 used the minimum, 5.8, and fifth percentile solely from the CPS-II cohort. The distribution's resulting lower/upper bounds for GBD 2019 were 2.4 and 5.9. This has not changed since GBD 2015.

Relative risks and population attributable fractions.

We create one set of cause-specific risk curves for household air pollution and ambient air pollution as two different sources of PM2.5. In GBD 2017, we estimated the particulate matter attributable burden of disease based on the relation of long-term exposure to PM2.5 with ischemic heart disease, stroke (ischemic and hemorrhagic), COPD, lung cancer, acute lower respiratory infection, and Type II Diabetes. In GBD 2019, we added adverse birth outcomes, including low birthweight and short gestation. Because these are already risk factors (and not outcomes) in the 82 GBD, we performed a mediation analysis, in which a proportion of the burden attributable to low birthweight and short gestation was attributed to PM2.5 pollution. For the six non-mediated outcomes, we used results from cohort and case-control studies of ambient PM2.5 pollution, cohort studies, case-control studies, randomized controlled trials of household use of solid fuel for cooking, and cohort and case-control studies of secondhand smoke. For the first time in GBD 2019, we no longer use active smoking data in the risk curves.

RESULTS

Table S1. Libya burden of disease related to fine particulate matter smaller than 2.5um (PM 2.5), 1990, 2000, 2010, and 2019, by year, and diagnosis, rate per 100,000, age-standardized.

	**Year**	**1990**				**2019**			
	Measure	Deaths	DALYs	YLLs	YLDs	Deaths	DALYs	**YLLs**	**YLDs**
**All causes**	Value (Lower-Upper)	62 (40-85)	1,779 (1,170-2,442)	1,623 (1,067-2,214)	157 (94-229)	70 (50-96)	1,930 (1,402-2,546)	1,612 (1,158-2,207)	317 (205-439)
**Ischemic heart disease**	Value (Lower-Upper)	30 (19-43)	710 (445-1,021)	696 (437-1,004)	14 (8-23)	36 (25-52)	853 (604-1,218)	832 (582-1,189)	22 (13-33)
**Ischemic Stroke**	Value (Lower-Upper)	8 (5-13)	183 (115-274)	148 (90-226)	35 (20-52)	12 (8-18)	299 (201-418)	239 (154-348)	60 (40-83)
**Type 2 Diabetes**	Value (Lower-Upper)	2 (1-3)	106 (61-154)	44 (25-66)	62 (32-99)	4 (3-6)	252 (162-357)	83 (54-122)	169 (99-255)
**Intracerebral hemorrhage**	Value (Lower-Upper)	5 (3-8)	146 (89-212)	137 (83-202)	8 (5-13)	4 (2-5)	107 (69-155)	98 (61-147)	9 (6-12)
**Chronic Obstructive Pulmonary Disease**	Value (Lower-Upper)	4 (2-6)	94 (61-135)	63 (38-95)	31 (20-45)	4 (3-6)	125 (78-181)	73 (42-110)	52 (33-73)
**Total cancers**	Value (Lower-Upper)	4 (2-6)	89 (49-133)	88 (49-132)	1 (0-1)	4 (3-6)	104 (63-150)	103 (62-149)	1 (1-1)
**Tracheal, bronchus, lung cancers**	Value (Lower-Upper)	4 (2-6)	89 (49-133)	88 (49-132)	1 (0-1)	4 (3-6)	104 (63-150)	103 (62-149)	1 (1-1)
**Lower respiratory infections**	Value (Lower-Upper)	4 (3-7)	163 (93-253)	161 (92-250)	2 (1-3)	4 (2-6)	84 (47-136)	82 (46-134)	1 (1-3)
**Neonatal preterm birth**	Value (Lower-Upper)	2 (1-3)	168 (70-281)	168 (70-281)	0 (0-0)	1 (0-1)	46 (17-80)	46 (17-80)	0 (0-0)
**Other neonatal disorders**	Value (Lower-Upper)	1 (0-1)	55 (22-96)	55 (22-96)	0 (0-0)	0 (0-1)	28 (8-55)	28 (8-55)	0 (0-0)
**Neonatal Encephalopathy due to birth asphyxia & trauma**	Value (Lower-Upper)	0 (0-1)	26 (9-51)	26 (9-51)	0 (0-0)	0 (0-0)	7 (2-13)	7 (2-13)	0 (0-0)
**Subarachnoid hemorrhage**	Value (Lower-Upper)	1 (0-1)	26 (15-41)	24 (13-38)	3 (1-4)	1 (0-1)	18 (11-28)	15 (9-24)	3 (2-5)

**Neonatal sepsis & other neonatal infections**	Value (Lower-Upper)	0 (0-0)	6 (2-11)	6 (2-11)	0 (0-0)	0 (0-0)	4 (2-9)	4 (2-9)	0 (0-0)
**Diarrheal diseases**	Value (Lower-Upper)	0 (0-0)	3 (1-8)	3 (1-8)	0 (0-0)	0 (0-0)	1 (0-1)	1 (0-1)	0 (0-0)
**Hemolytic diseases & other neonatal jaundice**	Value (Lower-Upper)	0 (0-0)	3 (1-6)	3 (1-6)	0 (0-0)	0 (0-0)	0 (0-1)	0 (0-1)	0 (0-0)
**Meningitis**	Value (Lower-Upper)	0 (0-0)	1 (0-2)	1 (0-2)	0 (0-0)	0 (0-0)	0 (0-0)	0 (0-0)	0 (0-0)
**Upper respiratory infections**	Value (Lower-Upper)	0 (0-0)	0 (0-0)	0 (0-0)	0 (0-0)	0 (0-0)	0 (0-0)	0 (0-0)	0 (0-0)
**Sudden infant death syndrome**	Value (Lower-Upper)	0 (0-0)	1 (0-2)	1 (0-2)	N/A	0 (0-0)	0 (0-0)	0 (0-0)	N/A
**Otitis media**	Value (Lower-Upper)	0 (0-0)	0 (0-0)	0 (0-0)	0 (0-0)	0 (0-0)	0 (0-0)	0 (0-0)	0 (0-0)
**Encephalitis**	Value (Lower-Upper)	0 (0-0)	0 (0-0)	0 (0-0)	0 (0-0)	0 (0-0)	0 (0-0)	0 (0-0)	0 (0-0)

*DALYs: disability-adjusted life years; YLLs: Years of life lost; YLDs: Years lived with disability.

Figure S1. Deaths (a) and Disability-adjusted life years, DALYs (b) attributable to PM2.5 Libya in 2019, by age and both sexes (per 100,000).

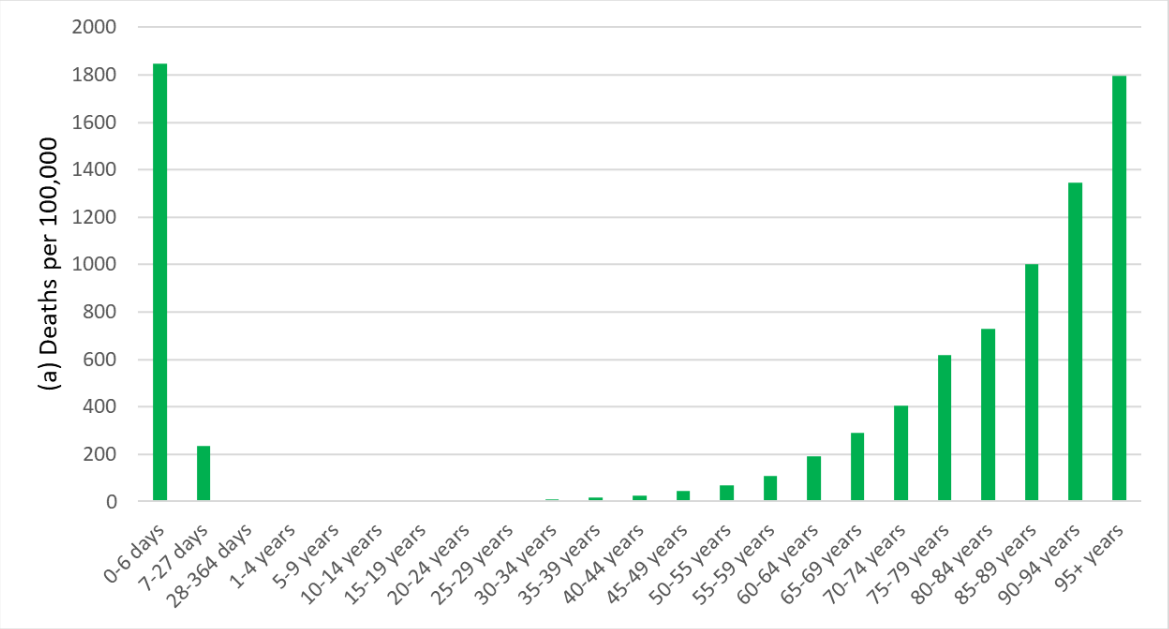

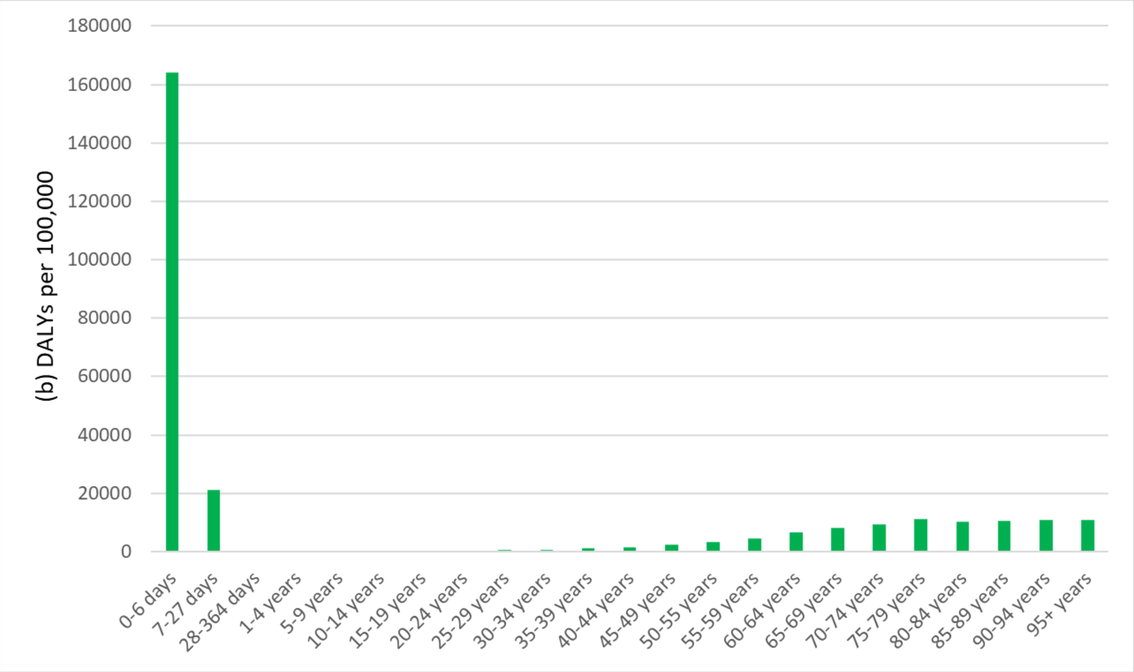

Figure S2. Annual deaths (all-cause) attributable to fine particulate matter smaller than 2.5μm (PM2.5) per 100,000 people (both sexes, ages-standardized).

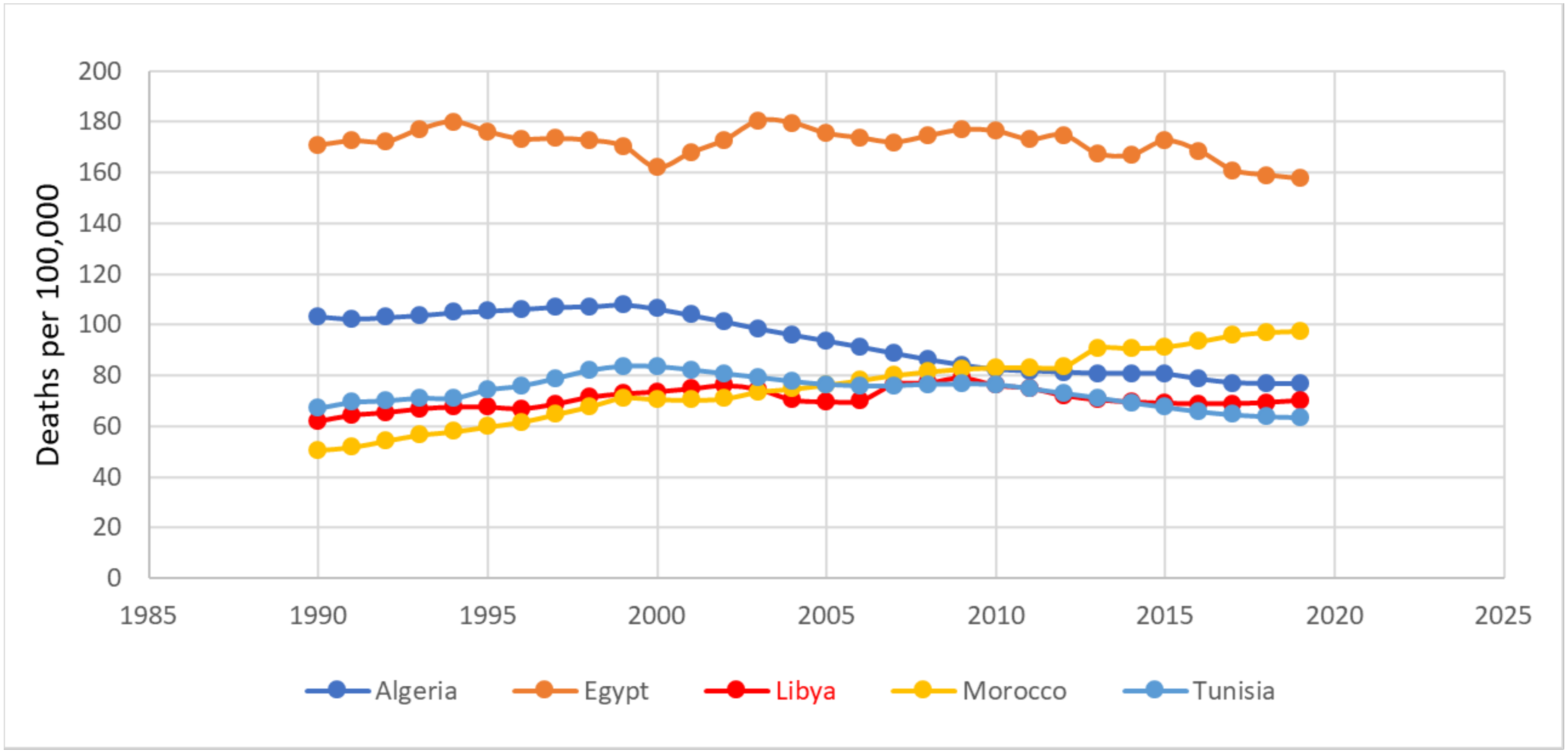

Figure S3. Disability-adjusted life years (DALYs) attributable to fine particulate matter smaller than 2.5µm (PM2.5) per 100,000 people (both sexes, ages-standardized).

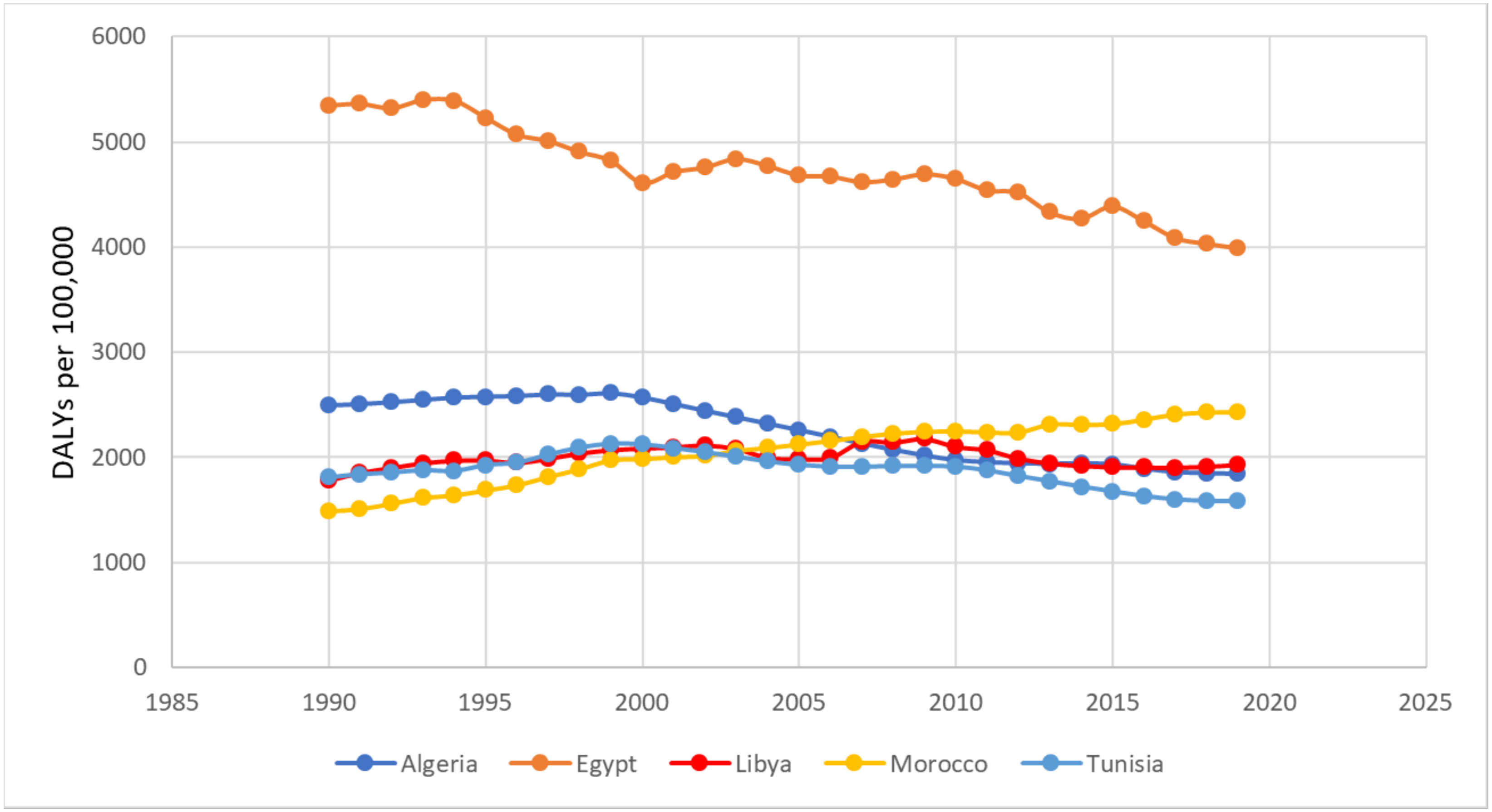

Figure S4. Treemap of (a) cause-specific mortality and (b) disability-adjusted life years (DALYs) attributed to PM2.5 in Libya, 2019, per 100,000 people (males, age-standardized).

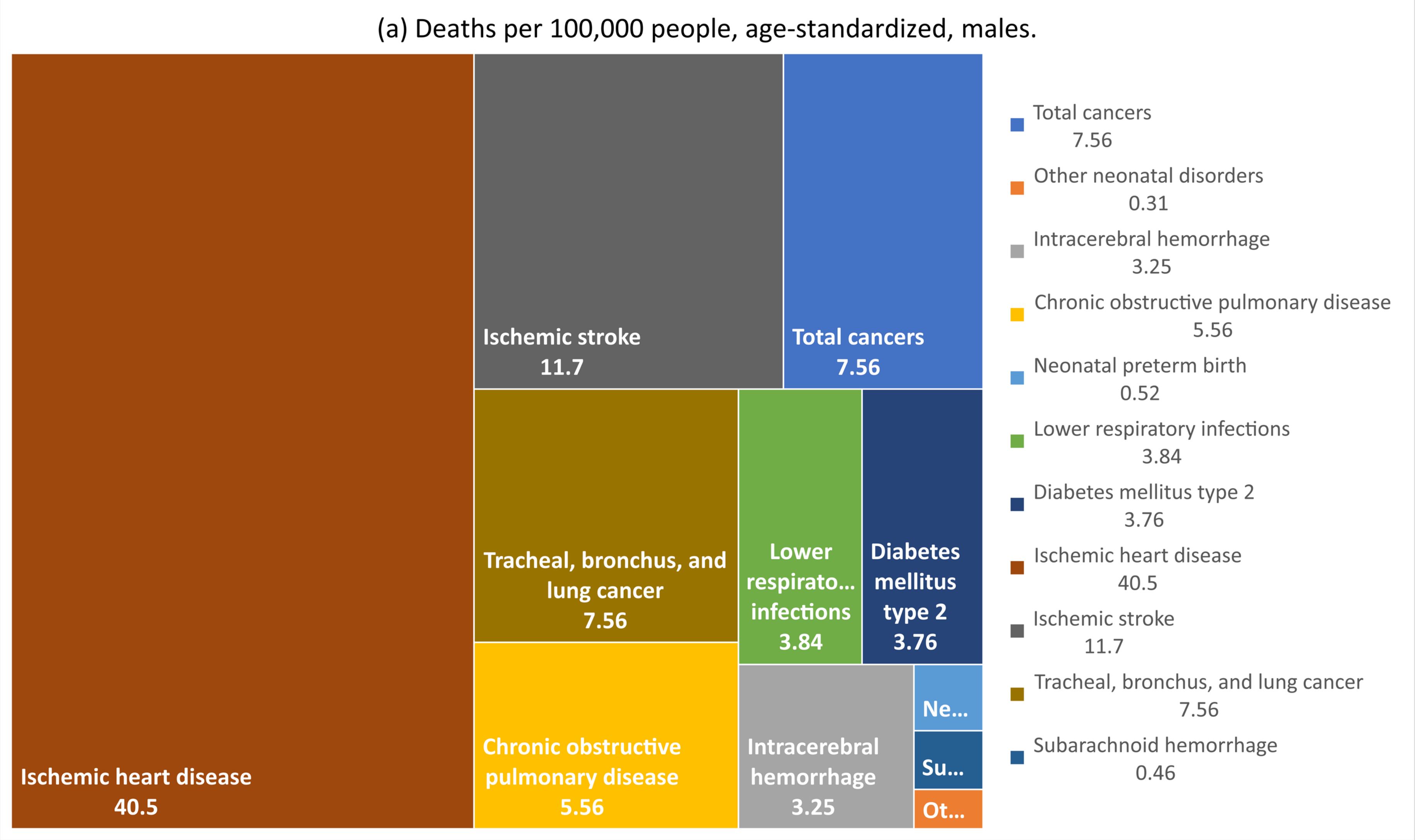

(b) DALYs per 100,000 people, age-standardized, males.

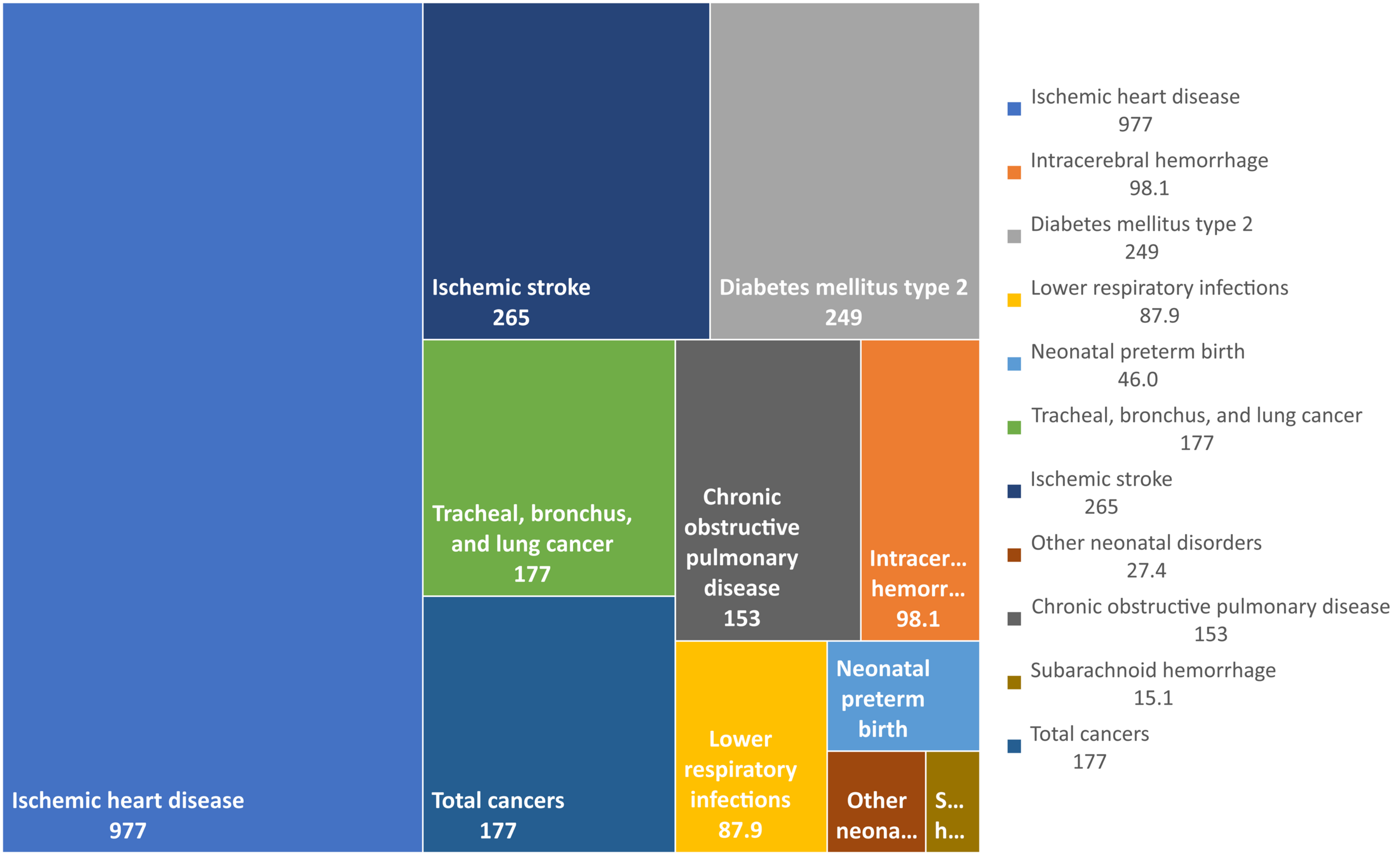

Figure S5. Treemap of (a) cause-specific mortality and (b) disability-adjusted life years (DALYs) attributed to PM2.5 in Libya, 2019, per 100,000 people (females, age-standardized).

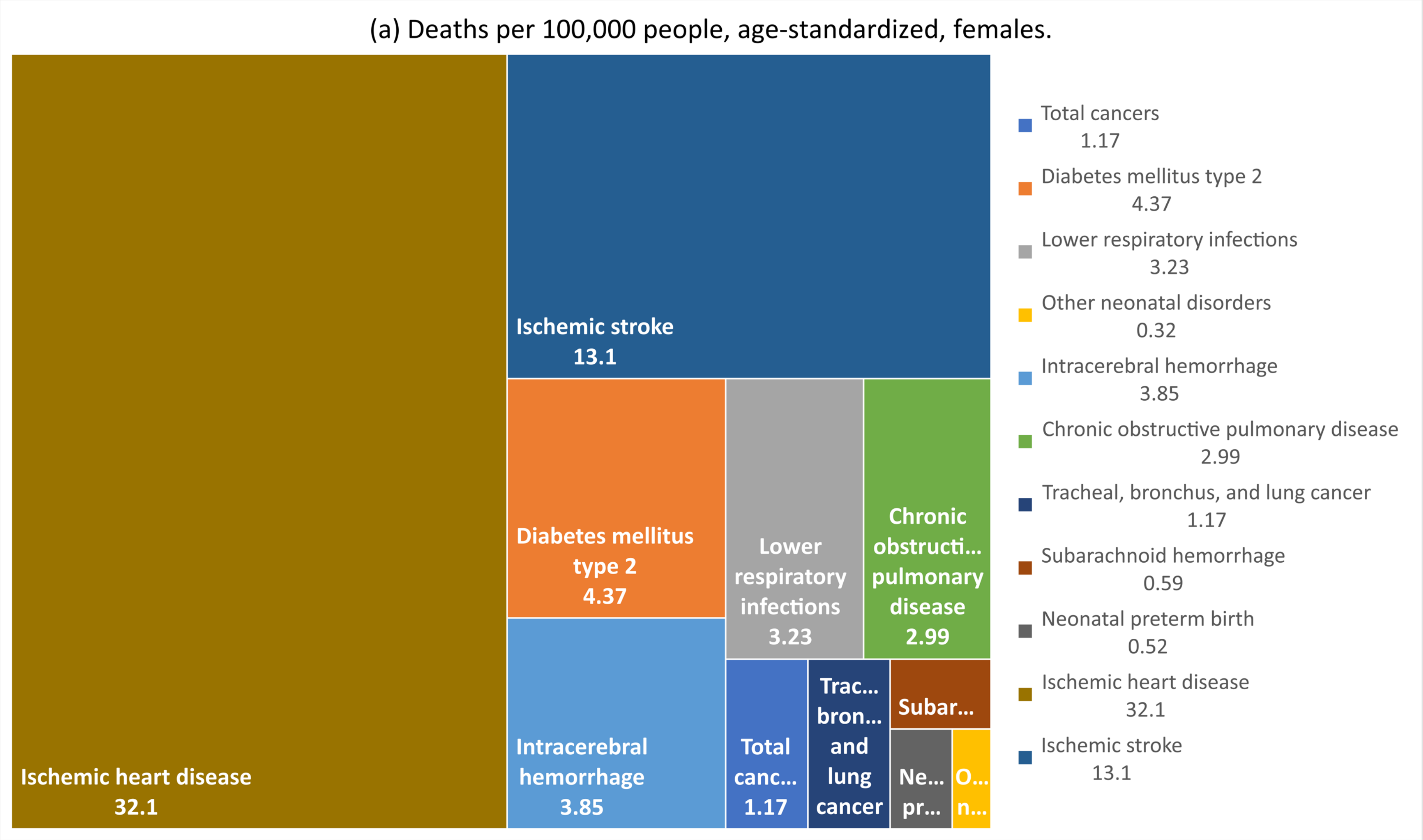

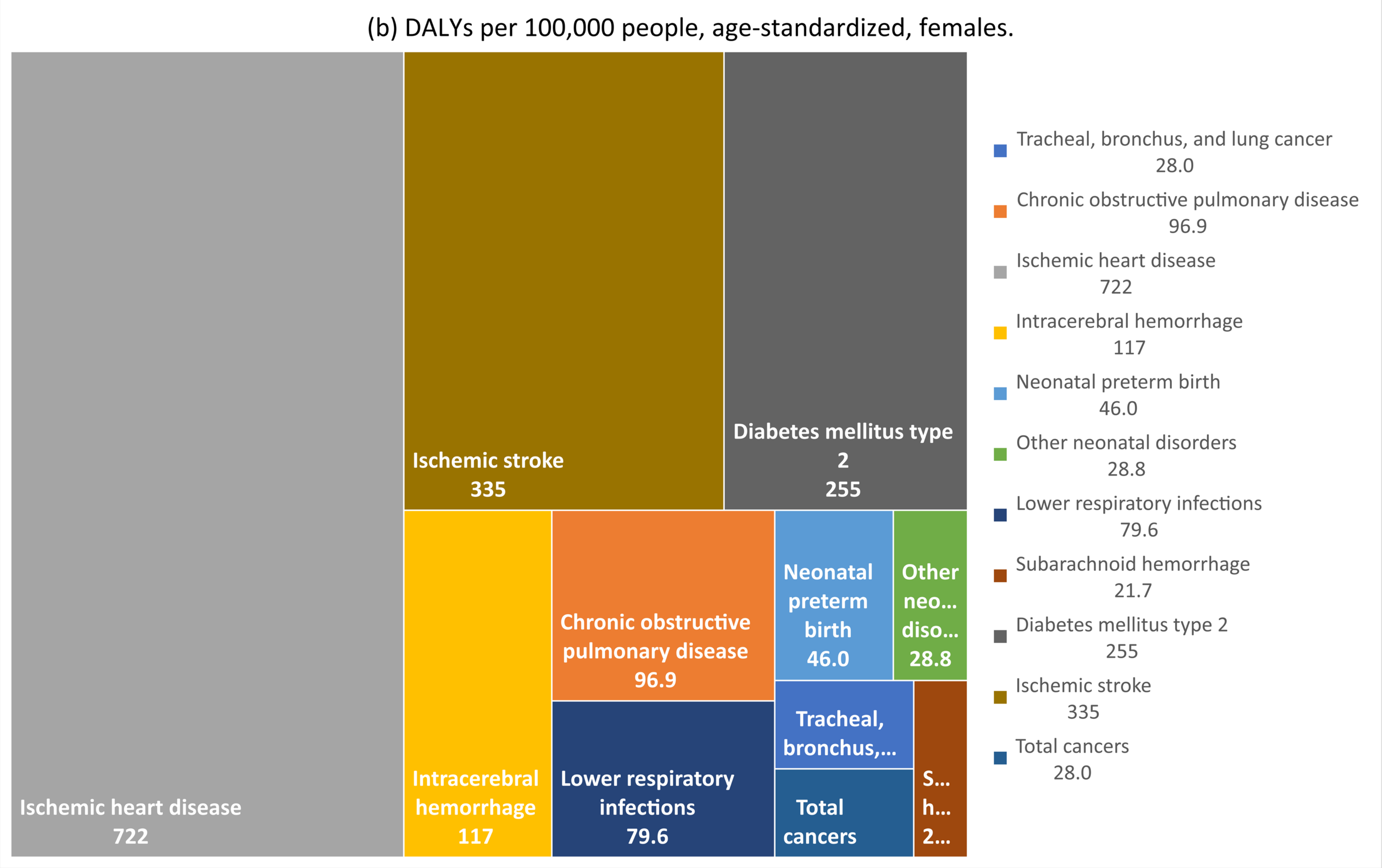
(b) DALYs per 100,000 people, age-standardized, females.
Ischemic heart disease 722
Ischemic stroke 335
Diabetes mellitus type 2 255
Intracerebral hemorrhage 117
Chronic obstructive pulmonary disease 96.9
Lower respiratory infections 79.6
Neonatal preterm birth 46.0
Other neo... diso... 28.8
Tracheal, bronchus,...
Total cancers
S... h... 2...
Tracheal, bronchus, and lung cancer 28.0
Chronic obstructive pulmonary disease 96.9
Ischemic heart disease 722
Intracerebral hemorrhage 117
Neonatal preterm birth 46.0
Other neonatal disorders 28.8
Lower respiratory infections 79.6
Subarachnoid hemorrhage 21.7
Diabetes mellitus type 2 255
Ischemic stroke 335
Total cancers 28.0
